# Evidence for tritium persistence as organically bound forms in river sediments since the past nuclear weapon tests

**DOI:** 10.1038/s41598-019-47821-1

**Published:** 2019-08-07

**Authors:** Frédérique Eyrolle, Yoann Copard, Hugo Lepage, Loic Ducros, Amandine Morereau, Cécile Grosbois, Catherine Cossonnet, Rodolfo Gurriaran, Shawn Booth, Marc Desmet

**Affiliations:** 1 Institut de Radioprotection et de Sureté Nucléaire (IRSN), PSE-ENV, SRTE/LRTA, SEREN/LEREN, SAME/LMRE, BP 3, 13115 Saint-Paul-lez-Durance, France; 2 University of Rouen-Normandie, UMR CNRS 6143 M2C, 76821 Mont Saint Aignan, France; 3 University of Tours, EA 6293 Géohydrosystèmes continentaux, Tours Cedex, France

**Keywords:** Environmental impact, Element cycles

## Abstract

Tritium of artificial origin was initially introduced to the environment from the global atmospheric fallout after nuclear weapons tests. Its level was increased in rainwaters by a factor 1000 during peak emissions in 1963 within the whole northern hemisphere. Here we demonstrate that tritium from global atmospheric fallout stored in sedimentary reservoir for decades as organically bound forms in recalcitrant organic matter while tritium released by nuclear industries in rivers escape from such storages. Additionally, we highlight that organically bound tritium concentrations in riverine sediments culminate several years after peaking emission in the atmosphere due to the transit time of organic matter from soils to river systems. These results were acquired by measuring both free and bound forms of tritium in a 70 year old sedimentary archive cored in the Loire river basin (France). Such tritium storages, assumed to be formed at the global scale, as well as the decadal time lag of tritium contamination levels between atmosphere and river systems have never been demonstrated until now. Our results bring new lights on tritium persistence and dynamics within the environment and demonstrate that sedimentary reservoir constitute both tritium sinks and potential delayed sources of mobile and bioavailable tritium for freshwaters and living organisms decades after atmospheric contamination.

## Introduction

## What we currently Know on Environmental Behavior of Tritium and what we Looked for

Tritium is a rare isotope of hydrogen and its only radioactive form. Tritium is a low-energy beta emitter, having limited radiotoxicity, and disappears from the environment after several decades by radioactive decay (physical half-life of 12.31 years). Despite the difference in mass between tritium and hydrogen, both occur in the same physicochemical forms. Hydrogen and its isotopes are ubiquitous in the environment, are particularly mobile and highly exchangeable both within and between abiotic and biotic components. Although the most common form of tritium in the natural environment is tritiated water (HTO), tritium, like hydrogen, is also bound to naturally occurring organic matter (OM) in the biosphere (organically bound tritium or OBT) and is then involved both in the water cycle and indirectly in the carbon biogeochemical cycle. While part of OBT (<30%) is easily exchangeable with water molecules in the surrounding environment, most OBT is sequestered long term. Its persistence within OM mainly depends on biodegradation rates of involved organic compounds^1^. In soils and river systems, biodegradation processes affecting particulate OBT are expected to produce dissolved OBT and subsequently free mobile hydrogen that can rapidly oxidize towards tritiated water molecules. According to biogeochemical equilibrium describing most natural environmental systems, concentrations of free and bound forms of tritium are expected to be similar and therefore, ratios of OBT/HTO would be close to unity. Nevertheless, any perturbation due to artificial inputs of tritium into the environment can alter this equilibrium since water masses are recycled faster than OM. These processes are assumed today to explain most OBT/HTO disequilibrium widely observed by several authors in various environmental compartments such as terrestrial plants^2–4^, aquatic plants^5,6^, soils^7,8^ and river sediments collected far from the influence of releases from nuclear facilities^5,9^. Among other hypotheses such as isotopic fractionation or hypothesis dealing with tritium bioaccumulation^10^, current consensual explanation for OBT/HTO disequilibrium is that tritium integrated into the OM would persist for the long term while the free forms rapidly exchange with the surrounding environment^1^.

Over the nuclear era, tritium contents in the atmosphere were significantly modified leading to drastic perturbation of the natural equilibrium of this element within environmental compartments at the global scale. Past atmospheric nuclear weapons tests performed from 1945 to 1981 have introduced around 200 times more tritium into the atmosphere (200 10^18^ Bq) than the global natural content at equilibrium (1 10^18^ Bq), and, during peak emissions in 1963, tritium content in rainwater was increased by a factor of nearly 1000 throughout the whole Northern hemisphere where most of the nuclear tests were performed^11–15^. Current tritium content in the atmosphere has almost decreased towards pre-bomb tests levels with 90% of tritium from nuclear tests having being stored in the oceans, and less than 5% of the amount produced at that time still remain^16^. Recent studies indicate that natural processes related to climate change would be responsible for the remobilization of tritium initially trapped in freshwater reservoirs since the global fallout from nuclear weapon tests: permafrost thaw due to warming temperatures would currently remobilize initially trapped HTO towards lakes and rivers^17^; glacier melting is expected to enhance tritium concentrations in surface and underground waters due to its remobilization from such reservoirs where tritium was buried^18^. Inputs from glacier melting are especially of concern nowadays because the mean residence time of waters in such reservoirs is close to 60 years. Thus, contaminated waters from nuclear weapon tests are potentially being remobilized since the beginning of the 2010s. Residual tritium activities from peak emissions in 1963 have declined today by almost 96% due to the radioactive decay of tritium. Nevertheless, theoretical residual levels would still range today from 10 to 30 Bq/L, without considering any dilution processes. Those levels are significantly higher than current tritium contents in rains and river waters (<1 Bq/L).

In addition to nuclear testing that affected natural contents of tritium at the global scale, human activities are also responsible for tritium emission in the atmosphere and in rivers at regional and/or local scales all around the world. In the southern part of the coastal region of Fukushima prefecture (Japan), HTO concentrations measured in wells near the damaged Fukushima-Daiichi nuclear power plant (FDNPP) and in rainwater were significantly increased during several weeks after the accident^19–22^ and huge volumes of tritiated water produced by the damaged FDNPP are still stored in containers close to the power plant. Tritium is also the main artificial radionuclide currently released in waters by nuclear power plants around the world and the expected evolution of nuclear civil technology (e.g. experimental fusion reactor) would produce and release additional tritium into the environment in the coming decades^23^. Hot spots of tritium contamination in various reservoirs (water, soils, sediments, organisms) are also observed more or less locally. These are sourced to *(a)* areas where nuclear tests were performed such as Kazakhstan^24^ *(b)* areas with past intensive use of nuclear fuel (e.g. in Russia^25^, *(c)* factories producing tritium for worldwide uses such as the Savannah River laboratories in the United States^26,27^, *(d)* industries producing self-luminescent paints or labelled organic molecules for pharmaceutical research purposes^1,28,29^.

Even though tritium is strongly mobile in the environment through its HTO form and rapidly disperses and dilutes within water masses, the OBT form is frequently found in some reservoirs where OM is preserved such as in soils^7,8^ and in riverine, lacustrine or marine surface sediments^5,9,28,30^. Consequently, if such OBT storages exist, we can expect that biogeochemical processes in these reservoirs due to OM degradation would act as a delayed source of HTO and/or dissolved OBT for the atmosphere and hydrosphere. In this context, our work aimed at determining whether or not tritium can persist and be conserved for decades in its OBT form in such OM reservoirs. In this aim we focused our work on a riverine sedimentary archive collected in the Loire River (France).

## Results Acquired from our Work

### A well preserved 70 year old riverine sedimentary archive

**Figure 1:**
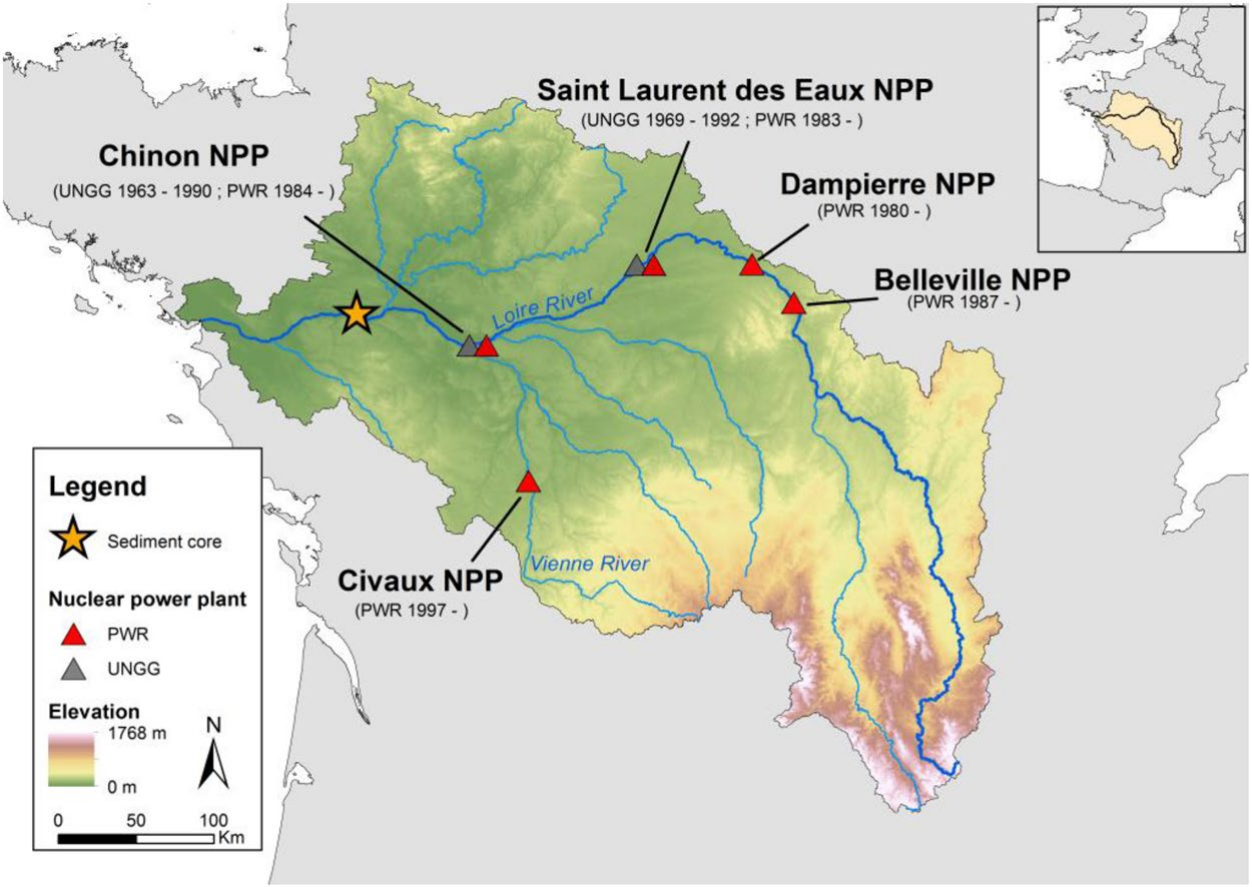
The sediment core was collected on 09/13/2016 on the right bank of an island in the Loire River (France) located downstream from nuclear power plants (NPP) which have released tritium since the beginning of their operation (PWR, pressurized water reactor; UNGG, natural uranium graphite gas reactor).

Riverine sediment archives are generally viewed as a recording of past mineral and organic sedimentary fluxes and associated contaminants. In order to test the hypothesis of tritium long-term (i.e. around 85 years) storage in such reservoirs, we measured OBT concentrations in a sedimentary core collected in 2016 from a river bank in one of the main nuclearized watersheds in Europe, the Loire basin in France (47°23′34″N, 0°51′23″E, Fig. 1). A sedimentary archive from this site was previously studied for trace metal temporal dynamics by^31^ and was expected to provide tritium fingerprints either from the liquid effluents released by nuclear power plants (NPP’s) over decades or from catchment erosion where tritium was initially trapped in terrestrial plants during photosynthesis. While the former inputs integrate aquatic particulate organic matter (aquatic POM) in river only, the latter would be carried by terrestrial POM transferred to the river due to surface processes (i.e. erosion). Hence, we first discriminated the POM nature and origin (i.e. aquatic vs terrestrial) by using biogeochemical and palynofacies analyses and then determined HTO and OBT concentrations within the sediment core. Results from the sediment age model using ^137^Cs and plutonium isotopes show that the 1 m long archive covers almost the last seven decades (1950–2016) with rather constant sedimentation rates over two main periods 1963–1986 (2.4 ± 0.2 cm/y) and 1986–2016 (1.3 ± 0.2 cm/y) as expected from^31^ (Supplementary Information – Fig. A).

### What is the origin of particulate organic matter stored in the archive?

**Figure 2:**
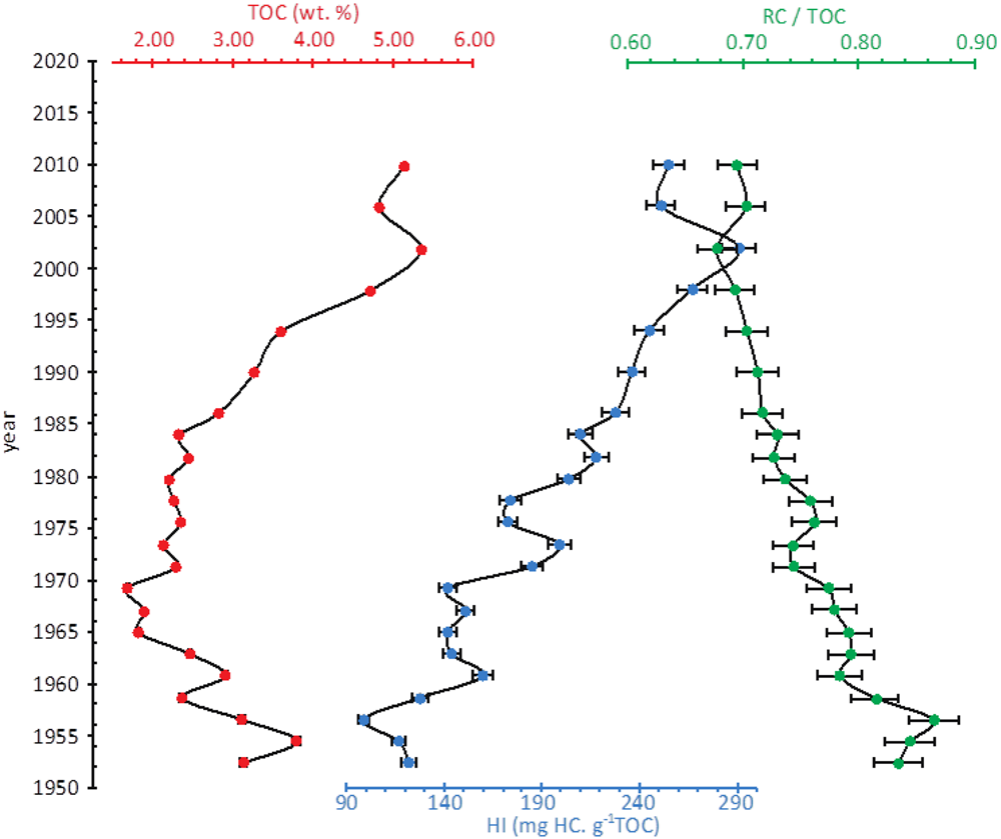
As seen with the hydrogen content (HI values), sedimentary POM mainly sources from the Loire catchments while its recalcitrant properties tends to become stronger (RC/TOC signals) with the age of sediments deposition; This change in POM nature is accompanied by a progressive and classical early POM process, named diagenesis, highlighted by the TOC and the HI signals.

**Figure 3:**
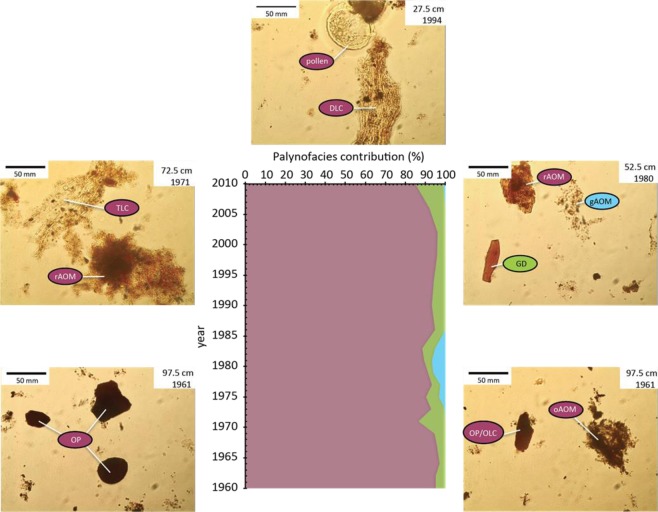
Pictures of various OM particles preserved in the archive at 27.5, 52.5, 72.5 and 97.5 cm depths with their associated age, see text for the explanation of the acronyms; each recognized particles are labeled of a color (purple, green, blue) outlining the OM origin (respectively: terrestrial, aquatic/riverbank, algal s.s.). A seen in the diagram, the terrestrial POM from the Loire catchment is the dominant fraction of sedimentary POM while the aquatic POM can be divided into two categories: the algal particles and the POM probably sourced from the riverbank and processed under a hydromorphic context. Note that the high aquatic productivity due to eutrophic conditions prevalent between 1978 and 1986 is observed.

POM was characterized to provide the different sources of particulate organic matter (POM) trapped in the archive by using classical global organic geochemistry analyses including the measurement of the hydrogen index (HI), total organic carbon (TOC), and refractory carbon (RC) contents. These parameters give information on the levels of degradation and potential origin of POM. Optical properties (palynofacies signatures) of POM were also investigated in order to discriminate between aquatic and terrestrial origins. From the top to the bottom of the sedimentary core, we observed a decrease in the richness of hydrogen content (hydrogen index, HI) accompanied by an increase in the proportion of refractory carbon (RC/TOC ratio; Fig. 2). In fluvial archives, the sources of POM is either aquatic or terrestrial, but, as seen frequently, the aquatic POM enriched in lipids is preferentially degraded during early processes (e.g.^32^). Interpretation of the several parameters from bulk organic geochemistry indicates that the preferential degradation of aquatic POM may occur between the top of the archive and 87.5 cm depth (post 1965), while the terrestrial fraction, rather depleted in lipid components, would have been relatively well preserved in the sediments and remain dominant in the deeper areas (prior to 1965). Palynofacies analyses show that the detrital fraction consists mainly of (i) low (translucent ligno-cellulosic debris - TLC) to significantly degraded ligno-cellulosic debris (DLC), (ii) weathered soil POM (reddish amorphous OM - rAOM), and to a lesser extent (iii) cuticles, membranes and spores without any significant transformation of the POM (Fig. 3). The fresh or slightly degraded particles progressively decline, in depth, to the benefit of opacified particles (OP) indicative of some significant states of the degradation of POM (opaque amorphous POM (oAOM), opaque debris (OD), and opaque lignocellulosic particles (OLC)). The optical study of quantitative palynofacies fully confirms the results from global organic geochemistry since the terrestrial POM contribution represents at least 85 wt.% of the POM content (Fig. 3). Aquatic POM particles are therefore in a minor proportion and essentially consist in gray amorphous OM (gAOM) from aquatic primary productivity, and gelified particles (GD) transformed under a hydromorphic context which may indicate a riverbank origin (e.g.^33^) (Fig. 3). We explain the relatively low but significant occurrence of algal POM in the sediments section 37.5–57.5 cm (i.e. between 1978 and 1986) by an increase in the aquatic organic matter productivity during this period which would have been important enough to prevent the total degradation of the POM quantities produced^33^. The well-known eutrophic conditions of the Loire River over 1978–1986 leading to a high aquatic primary production, fully reinforces our hypothesis^34^. The confrontation of bulk geochemical and optical analyses suggests that degradation processes lead to an increase in the proportions of more refractory organic material, i.e. stable, from the surface to the depth. We suggest that these last compounds originate from the evolved litter and organo-mineral soil horizons and are thus older than their date of deposit within the archive.

### Free and bound forms of tritium among the sedimentary profile

**Figure 4:**
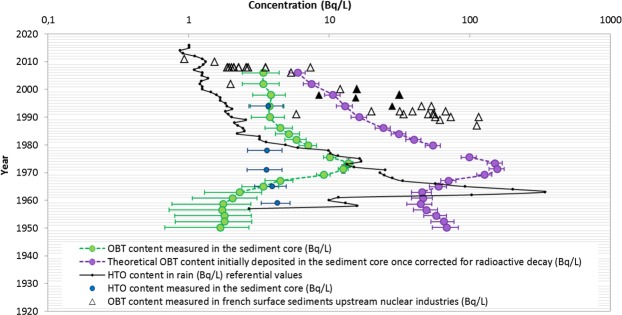
OBT concentrations measured in 2016 in the sediment core (green points) highlight a large amplitude symmetric peak culminating in 1973 ± 2 while HTO contents measured in rains (black points) display peaking concentrations in 1963 (Vienna, Austria, from IAEA Wiser data base, https://nucleus.iaea.org/Pages/GNIPR.aspx); OBT concentrations corrected from tritium radioactive decay at the theoretical time of sedimentary deposits (violin points) can be compared to OBT concentrations measured in surface sediments collected in French rivers upstream the influence of nuclear industries since the end of the 1980’s (triangles; black ones refer to the Loire River). HTO contents in the sediment core (water of freeze-drying dehydration) show almost constant values but higher than HTO concentrations in rains currently registered. All OBT and HTO contents are expressed in Bq/L for direct comparison purposes. https://nucleus.iaea.org/Pages/GNIPR.aspx).

Tritium analyses for the free (HTO) and bound forms (OBT) show that both forms are not at equilibrium. While HTO concentrations are almost constant without any trend with the depth (mean value 3.8 ± 0.3 Bq/L), OBT concentration profile shows a broad amplitude and symmetric peak during the early seventies (Fig. 4). OBT concentrations start to increase as early as 1963 ± 2 from relatively stable levels ranging from 1.7 ± 0.4 to 1.8 ± 0.3 Bq/L (average 1.75 ± 0.05 Bq/L) over the period 1950–1961, then rise to a maximum in 1973 ± 2 (13.7 ± 1.7 Bq/L), then gradually decrease to relatively constant values after the mid-1980s until recent years (averaging 3.6 ± 2.2 Bq/L). This scheme would directly reflect tritium footprints introduced during atmospheric deposition from past nuclear weapons tests, with a shift of one decade (peak emissions from nuclear tests in 1963).

## New Findings

### Only POM from terrestrial origin is preserved in such sedimentary archives

Riverine sediments collected onto alluvial margins are expected to contain mostly POM from terrestrial origin since those sedimentary sinks are formed mostly during flood events by the transportation of an enhanced proportion of particles from either bank or catchment surface erosion. POM characterization clearly indicates that the studied fluvial sedimentary record cannot be expected to retrace the industrial liquid tritium discharges due to its low aquatic POM contents but would rather restitute the memory of tritium incorporation in terrestrial plants as a result of atmospheric fallout from past nuclear weapon tests. Even though the studied archive would be characteristic of most of sedimentary reservoirs in large rivers, it is however not totally excluded that riverine sediments can preserve aquatic POM escaping from degradation processes either in water or during early diagenesis. Specifically, such preservation of aquatic POM could be promoted by factors either facilitating the preservation (e.g. increase of aquatic primary productivity, physical protection of labile OM by mineral matrices) or limiting its mineralization by *in situ* OM processes (e.g. formation of refractory OM from a labile precursor^32^. These pioneering results highlight that while tritium from atmospheric emission store in river sediments as bound forms to organic compounds originating from soils erosion in the catchment, tritium from nuclear releases in the river would escape from such storages in most cases.

### Tritium is preserved as organically bound forms in sedimentary reservoirs for decades

The results acquired give direct evidence of tritium preservation as OBT in sedimentary reservoirs over long periods of time, an important finding as this, while hypothesized, has never been demonstrated before to our knowledge. One of the most pioneering observations is the shift between the two tritium peaks registered on one hand within the sedimentary archive (1973 ± 2) and on the other hand in rainwaters (1963) (Fig. 4). This shift directly reflects the delayed transfer of terrestrial POM from the catchment to the river. This transfer time gap of POM (around one decade), from its production during the growth of terrestrial plants (photosynthesis) to its detrital export by rivers, is of the same order of magnitude as those evaluated by other authors throughout the French territory^5,9,35^. Interestingly, the symmetric shape of the OBT peak would suggest that POM in riverine sediments is a continuum of progressively decomposing organic compounds in soils as recently proposed by^36^ for soil OM. A further goal of OBT investigation within sediment cores would be the access to a new tool to estimate the age of such decomposing POM in river systems.

OBT contents measured along the core most probably include various proportion of E-OBT depending on both the nature of organic compounds involved and their degradation state. If we can consider rather constant levels for E-OBT with depth owing to equilibrium with surrounding mobile waters (E-OBT = HTO = 3.8 Bq/L) E-OBT/OBT ratios most probably differ along the profile due to both the variation of POM nature and characteristics without the possibility to be precisely estimated. The decrease in the richness of hydrogen content (HI) with depth would indicate a decreasing of exchangeable hydrogen then of E-OBT/OBT ratios along the profile. Even though E-OBT would dilute more or less the signal of total OBT we consider that this parameter cannot significantly modify the general trend of the OBT profile along the core, i.e. delayed OBT peak in 1973 ± 2. Further on, the peak OBT content within the archive (13.7 ± 1.7 Bq/L) is rather close to the theoretical residual tritium level in waters from peak emissions in 1963 after radioactive decay, i.e., from around 10 to 30 Bq/L, supporting additional evidence for OBT origin (Supplementary Information – Fig. B). Additionally, once corrected for the radioactive decay, OBT concentrations measured within the archive from 24.2 ± 3.0 Bq/L in 1986 to 6.0 ± 0.8 Bq/L in the more recent years (2006) approach those in surface sediments collected since the middle of the 80 s in various French Rivers upstream nuclear facilities, including the Loire River^5,9^ (Fig. 4). OBT contents in such surface sediments acquired in the upstream Loire River ranged from 8.4 ± 1.8 to 31.6 ± 2.8 Bq/L at the end of the nineties^5^ (Fig. 4). Those measured within the sedimentary archive over the same time period when corrected from radioactive decay are in the lower range of those last values, i.e. 10.6 ± 1.4 Bq/L. Interestingly, the gap in OBT concentrations between the two data sets, declining over time, probably reflect *(a)* a loss of OBT (mainly E-OBT) in the archive during the preferential degradation of the most recent POM and *(b)* an heterogeneous distribution of tritium among POM depending on the age of the various components (pre and post nuclear tests). It is likely that the differences between OBT concentrations measured in the sedimentary archive and those measured directly in surface sediments is partly related to POM degradation processes with time. Degradation of POM may lead to remobilization of OBT towards the water either as free forms (tritiated hydrogen or methane, HTO) or as bound forms with dissolved or colloidal organic molecules. Those mechanisms can lead to lower mass concentrations of OBT depending on the nature of the degraded compounds^37^. These processes, involving microorganisms, can also lead to OBT redistribution among the POM. Such mechanisms would also explain why low but detectable OBT levels were found in the early 50 s leading to unrealistic OBT levels over the pre-bomb tests period once corrected for radioactive decay.

The mobile form of tritium (HTO) is expected to quickly exchange within surrounding water bodies. HTO contents among the sediment core are significantly higher than those measured in rain waters (0.6 to 1.6 Bq/L in 2014^38^, but lower than those registered in the framework of radiological monitoring of the Loire River downstream NPP’s (from around 5 to 10 Bq/L^39^ or in the Loire estuary^40^. Even though HTO contents measured among the free water within the archive could conceptually display HTO enrichment from OBT degradation they most probably reflect HTO contents in the Loire River due to water mass connectivity during flooding or underground water exchanges. Finally, HTO contents within the archive would be the result of various dilution processes with rain waters and/or rapid exchange of water molecules with the atmosphere preventing the precise knowledge of HTO origins.

All results provide strong arguments for a conservative persistence of tritium for long term (decades) in POM reservoirs such as riverine sediments. Once again, while these mechanisms were previously hypothesized, they were never demonstrated before.

## Pioneering Research Results

Recent studies using time series from tritium monitoring in French rivers already reported significant disequilibrium between OBT and HTO contents in various river bed sediments collected upstream from NPPs in the upper catchment areas of French rivers^5,9^. It was hypothesized that tritium persistence within POM was imported from the watershed from past nuclear weapons tests. However, the data sets explored in these previous studies only covered the last twenty five years and had huge heterogeneity in terms of sample locations, sample treatments and analyses. Here we give direct evidence of such origin on a well preserved sedimentary core covering the whole nuclear era.

From a methodological point of view, our results are extremely pioneering, since OBT analyses were not technically available before the end of the eighties and consequently, the role of OBT in the long term storage of tritium was impossible to assess. Additionally, this is the first time that an OBT content profile within a sedimentary archive is presented that covers the entire nuclear era (more than sixty years) and reveals the potential route of tritium in continental surfaces at the global scale. To our knowledge, only three recent studies report OBT analyses along the profile of sedimentary archives. One of them was collected in the upper Rhône River (Eastern France)^1^ and the other one at the mouth of this same river^41^, but, in both of these cases, OBT levels recorded reflected a pronounced labelling reaching several thousand Bq/L related to technogenic tritium releases from watchmaking workshops located in the upstream catchments of the Rhône River (residues of tritiated paints, i.e. tritiated polystyrenes^1,9,42^. This technogenic OBT environmental labelling found in these archives masked the signal recorded in the archive studied here. In addition, a 44-year old sedimentary archive (1961–2015) collected in the Durance River (southeast France, Serre Ponçon dam) by our laboratory also revealed a significant OBT peak at the very end of the profile that was thought to be associated with the fallout from nuclear weapon tests^35^. The particularly erratic and strong sedimentation rates observed for this last archive prevented a precise dating of the OBT peak. However, the OBT levels associated with the peak of the Durance archive, of the order of 15 Bq/L, are similar to those observed in the archive studied here (12.5 ± 3.8 Bq/L) comforting evidence for tritium persistence from nuclear weapons tests as OBT in riverine sedimentary reservoirs at a large scale.

## Resulting Scientific Impact

Tritium atmospheric inputs onto catchments can be reconstructed by exploring riverine sedimentary cores. This original approach can be expected to be successfully applied in both riverine and marine coastal environment, such as in the case of Fukushima-Daiichi damaged NPP’s, in order to estimate tritium delivered after the accident and its origin (atmospheric deposits onto the catchment/liquid releases). This could offer new operational tools to reconstruct the radioecological impact from tritium deliveries in contaminated environments. Our results also directly demonstrate that tritium can be trapped within POM reservoirs for decades and would explain most of the current OBT/HTO disequilibrium within the various environmental compartments and components including living organisms. Apart from the scientific community closely involved with the environmental behavior of tritium, OBT analyses within sediment cores offer a new tool to estimate the age of recent POM in river systems to those working on naturally occurring organic compounds. Finally, POM reservoirs would act as a delayed source term of tritium for river and marine waters through POM transfer and degradation. OBT enriched particles and/or molecules as well as enhanced tritiated waters can then be expected close to riverine sedimentary reservoir several decades after initial contamination reinforcing delayed emission of tritium already expected from permafrost and glacier melting due to climate change.

## Significance

Tritium is a radionuclide that has increasingly affected the global environment since the dawn of the nuclear age and, if new fusion reactors are successful, they will contribute an additional source. Although previous work has shown that it is rapidly exchanged, diluted, and dispersed within water bodies, our study gives evidence that tritium persists for long periods in organic matter within reservoirs. We discovered fingerprints of tritium bound to organic compounds that were preserved since the nuclear weapon tests era in riverine sediments. Our work raises immediate concerns regarding post-accidental management of tritium emissions with direct application to the case of Fukushima (Japan). It also provides additional key knowledge for the future management of tritium releases from nuclear industries.

## Method Summary

### Location, sampling and samples

The sedimentary archive was collected near Montjean-sur-Loire (France) on 13 September 2016 (0°51′23.1″W–47°23′34.0″N; Altitude: 13 m) on the right bank of an island in the Loire River, just upstream of the estuary influence and downstream of the last NPP’s (Fig. 1**)**. Detailed descriptions of this coring site and of the Loire basin are reported in^31^. Sediment cores were sampled using a percussion corer (Cobra TT - SDEC) using tubes 45 mm in diameter and cut in 5 cm slices. Samples were freeze-dried under nitrogen flux before analyses.

### POM analyses

POM was characterized by using classical global organic geochemistry analyses including the measurement of the hydrogen index (HI), total organic carbon (TOC), and refractory carbon (RC) contents. POM was also characterized regarding to its palynofacies signatures. The analysis of the mass percentages of carbon and hydrogen, was performed using an elemental analyzer (Elementar). The freeze-dried sample were then decarbonated after an acid attack (a few ml of 1N HCl and then at least 24 hours on a sand bath at 80 °C) placed in tin foil, and then positioned in the analysis chain which includes an oven at 1000 °C and a reduction tube (600 °C). Particle size analyzes were performed using a laser diffraction granulometer (Coulter Beckman LS13320). The methodologies proposed to distinguish the different sources of POM consist in quantifying the POM content of the sediments by optical characterization (quantitative (palynofacies) and for bulk organic geochemistry (Rock-Eval 6 pyrolysis). The analysis of palynofacies discriminates the POM origin and the quantification of the different organic constituents. The analytical protocol aims to isolate the POM from the sample (2–5 g dried at 30 °C) by acid digestion (HCl/HF) of the mineral fraction^43^. The POM was then observed under light microscopy (Leica DMR XP) in transmitted light (objective x50) using a grid (10 μm mesh) where 200 elementary surfaces were considered to obtain a percentage of error lower than 5%^44^. The origin and the state of degradation are defined on the basis of morphological, textural and colorimetric criteria^45^. Since this method is surface-based, the quantitative palynofacies that allows access to the amount of POM (in mg/g) of the different classes defined by counting has been used^46^. The results obtained are coupled with the parameters resulting from the Rock-Eval 6 pyrolysis of the POM^47,48^ which does not require prior acid treatments to isolate the organic content and which delivers the organic carbon content of the sample (noted TOC, or POC), its richness in hydrogen in mg of hydrocarbons g^−1^ TOC (HI parameter) and its reactivity to supergene weathering (RC/TOC ratio, i.e., the residual organic carbon/total organic carbon ratio). These two methods thus provide the different sources of POM trapped in the archive as well as their refractory or labile nature in the face of surface processes (e.g. biodegradation, oxidation).

### Radionuclides analyses (^137^Cs, plutonium isotopes, HTO, OBT)

Once collected the core was transported to the laboratory where sediment samples were collected along the core and immediately stored at −25 °C. Then the samples were separately freeze dried under dehydrated nitrogen flux in order to avoid any atmospheric exchange before analyses. Water samples from freeze-drying were collected for HTO analyses. Solid samples from freeze drying were immediately stored in aluminized bags and under vacuum until HTO analyses in order to avoid contamination of the samples due to potential exchange of mobile hydrogen/tritium with the atmosphere of the laboratory. Water samples from freeze drying were immediately stored in dark glass containers (20 mL) stored at −4 °C in aluminized bags and under vacuum until analyses to limit exchanges with the surrounding atmosphere. These methodological cautions are necessary to insure the representativeness (quality) of the results obtained from both HTO and OBT analyses. In particular, these cautions avoid potential contamination between samples themselves through exchange with the atmosphere. HTO contents in the ambient water vapor of laboratories, where all samples were prepared, stored and analyzed, were monitored in order to control the absence of significant HTO contamination in the atmosphere of laboratories.

Gamma spectrometry analyses (^137^Cs) were performed by using Ge-HP type N or equivalent, coaxial detectors of at least 30% relative efficiency. The efficiency calibration was performed by Monte Carlo type simulations using GeSpeCor^49^ or Geant^50^. The activity was connected to the national primary standards through the use of accredited standard sources. The counting times used were 24 hours per sample. Plutonium isotope concentrations were measured by alpha spectrometry after radiochemistry steps. In the particular case of the downstream Loire River plutonium isotopes constitute chronological tracers as a consequence of liquid plutonium discharges in the Loire following the two accidents that occurred on the Saint-Laurent-des-Eaux NPP’s in 1969 and 1980. HTO analyzes in water samples from freeze-drying of sediment samples were performed by conventional liquid scintillation counting^51^. The decision threshold for this method is 0.65 Bq/L (10 mL, 17 h counting).

Organically bound tritium (OBT including E-OBT and NE-OBT) analyzes were performed by the ^3^He ingrowth method after placing the sample in a vacuum (<10^−5^ mBar) in order to extract residual gases and waters (^3^He, ^4^He, mobile hydrogen and tritium) and after storage under such a vacuum for 3 to 4 months depending on the expected OBT content of the sample and associated uncertainty required^52^. In contrast to other methods based on the combustion of the freeze dried sample in order to analyze tritium contents from the thermal degradation of organically bound forms (tritium in the water of combustion), the ^3^He ingrowth method is suitable for soil and sediment samples generally characterized by very low organic matter contents (not directly combustive, hydrogen contents less than 2% in the dry mass). Additionally, this latter method is the only one currently available to our knowledge to study such samples (soils and sediments) with low to very low OBT contents (less than 5 Bq/kg). This method determines the ^3^He levels produced by the decay of tritium (NE-OBT + E-OBT) contained in the sample after normalization of the values to ambient atmospheric levels (^3^He/^4^He) and correction of the radiogenic ^4^He levels contained in the sample (gaseous inclusions). The analysis results are expressed in Bq/kg_dry_. The conversion to Bq/L required the analysis of hydrogen content (%H). This last unit was necessary because it made it possible to address the carrier phase of tritium, that is to say, water, in hydrogen equivalent, and thereby allowed the comparison of the OBT levels with the HTO ones. As opposed to the method based on the tritium measurement in the water of combustion of the sample (directly expressed in Bq/L), the method using ^3^He mass spectrometry measure tritium content in the dry mass of the sample (Bq/kg_dry_); thus, total %H per dry mass of the samples (exchangeable and not exchangeable hydrogen) are measured in aliquots in order to convert Bq/kg in Bq/L by using the following equation: HTO (in Bq/L) = HTO (in Bq/kg_dry_) × (2/18) × H (in % of dry mass) × 100. OBT contents were not measured within the top layers of the archive (2006–2016) due to an unavailability of a sufficient quantity of sediment for analyses.

### Sediment core dating

Radiocesium concentrations with depth highlight two main peaks at 92.5 cm and 37.5 cm depth (Supplementary Information - Fig. A). These peaks are associated to peak radioactive emissions from atmospheric nuclear tests in 1963 and to fallout from the Chernobyl accident in 1986. This last peak is preceded by increasing concentrations from 67.5 cm of depth to 37.5 cm testifying to the discharges made by the NPPs gradually implanted on the Loire basin since 1957 (UNGG then PWR of Chinon) then from the beginning of the years 1980 for the WPR. Activity ratios ^238^Pu/^239+240^Pu also show two maximums, one at 77.5 cm depth (0.06 ± 0.01), the other at 52.5 cm (0.08 ± 0.01), located between the two peaks of ^137^Cs. In the absence of an industrial source, plutonium isotopes are the result of atmospheric fallout from surface nuclear tests (1945–1980) and atmospheric deposition related to the explosion of the Transit 5 BN-3 satellite over the Indian Ocean (1964). The activity ratios significantly higher than those characterizing the atmospheric reference values are associated with the accidental liquid plutonium discharges that occurred in 1969 and 1980 on the Saint-Laurent-Les-eaux NPP’s. Thus, peaks of ^238^Pu/^239+240^Pu activity ratio constitute, in addition to the peaks of ^137^Cs, two additional chronological markers for dating the archive. From these chronological landmarks (1963, 1669, 1980, 1986 and 2016), a simplified age model was applied connecting years and depths. Uncertainties on years are estimated to ±2 years.

## Supplementary information

SUPPLEMENTARY INFORMATION
