# Supplementary material for: Evidence for tritium persistence as organically bound forms in river sediments since the past nuclear weapon tests
Source: Sci Rep. 2019 Aug 7;9:11487. doi: 10.1038/s41598-019-47821-1 (PMC6686018; doi:10.1038/s41598-019-47821-1)
Supplement: Supplementary file 1 — SUPPLEMENTARY INFORMATION [file 41598_2019_47821_MOESM1_ESM.pdf]

SUPPLEMENTARY INFORMATION: Evidence for tritium persistence as organically bound forms in river sediments since the past nuclear weapon tests.

Authors: Frédérique Eyrolle, Yoann Copard, Hugo Lepage, Loic Ducros, Amandine Morereau, Cécile Grosbois, Catherine Cossonnet, Rodolfo Gurriaran, Shawn Booth, Marc Desmet.

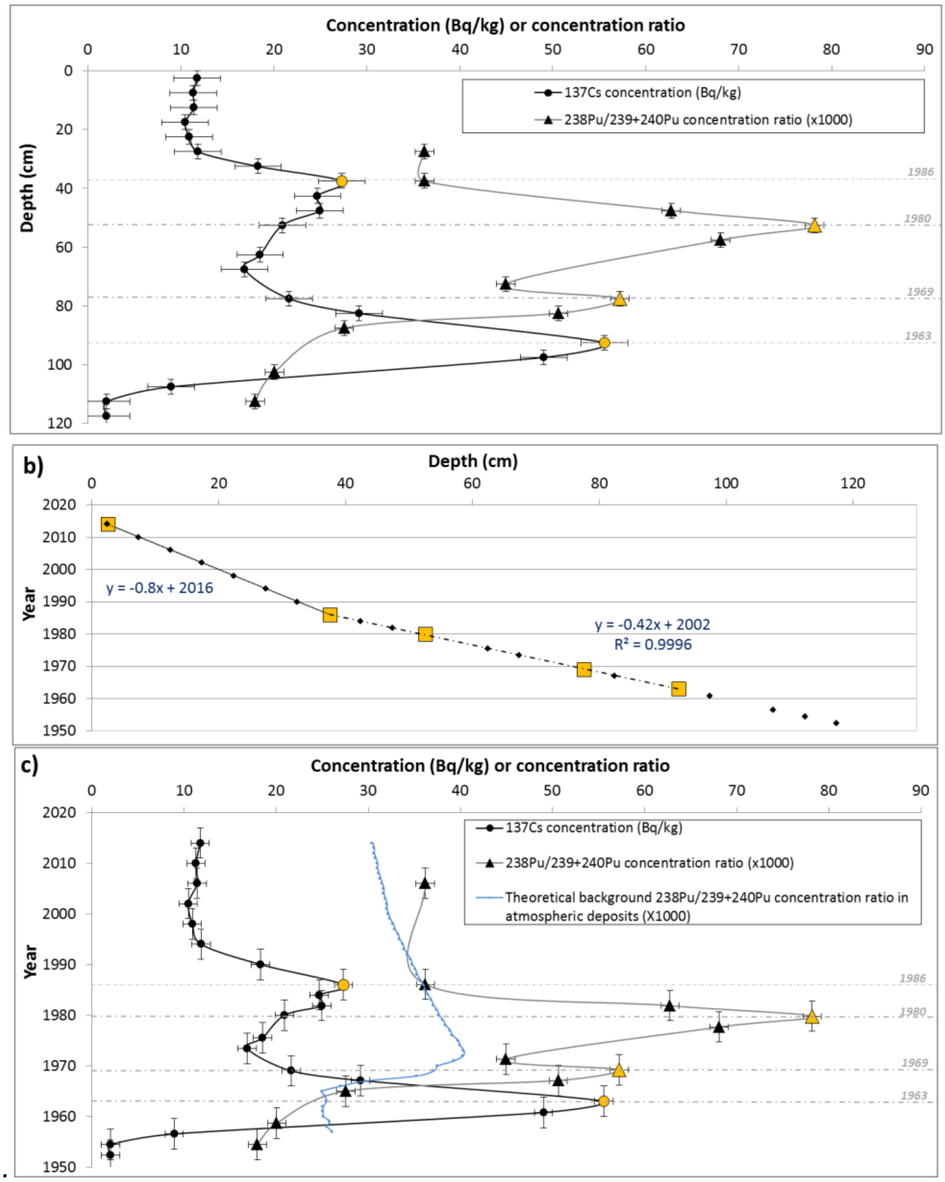

Figure A: Sediment core dating: a) ^{137}Cs concentration (Bq/kg) and $^{238}Pu/^{239+240}Pu$ concentration ratio (x1000) with depth. The two peaks of ^{137}Cs in 1963 (due to peak emissions from nuclear weapons tests in the atmosphere), then in 1986 (due to atmospheric fallout from the Chernobyl accident in Europe), and the two peaks of $^{238}Pu/^{239+240}Pu$ activity ratio in 1969 and 1980 (occurring after the Saint-Laurent-des-Eaux nuclear reactor accidents) were used as chronological references (yellow points); b) These references were used to derived two linear models connecting age (year) and depth (cm), below and above 37.5 cm (before and after 1986) reflecting distinct sedimentation rates among these periods; c) ^{137}Cs concentration (Bq/kg) and $^{238}Pu/^{239+240}Pu$ concentration ratio (x1000) over time. Years associated to the various depths were calculated from the linear models. Theoretical background $^{238}Pu/^{239+240}Pu$ concentration ratios over time from both global atmospheric fallout from surface nuclear weapon tests and satellite Transit 5BN-3 explosion over the Indian Ocean in 1964 (Northern hemisphere, latitude of France, from (1) are reported on (c) in order to highlight $^{238}Pu/^{239+240}Pu$ concentration ratio peaks originating from the two NPP's nuclear accidents. Background $^{238}Pu/^{239+240}Pu$ concentration ratio values peaked in early 1973 over France due to air mass transfer and dilution.

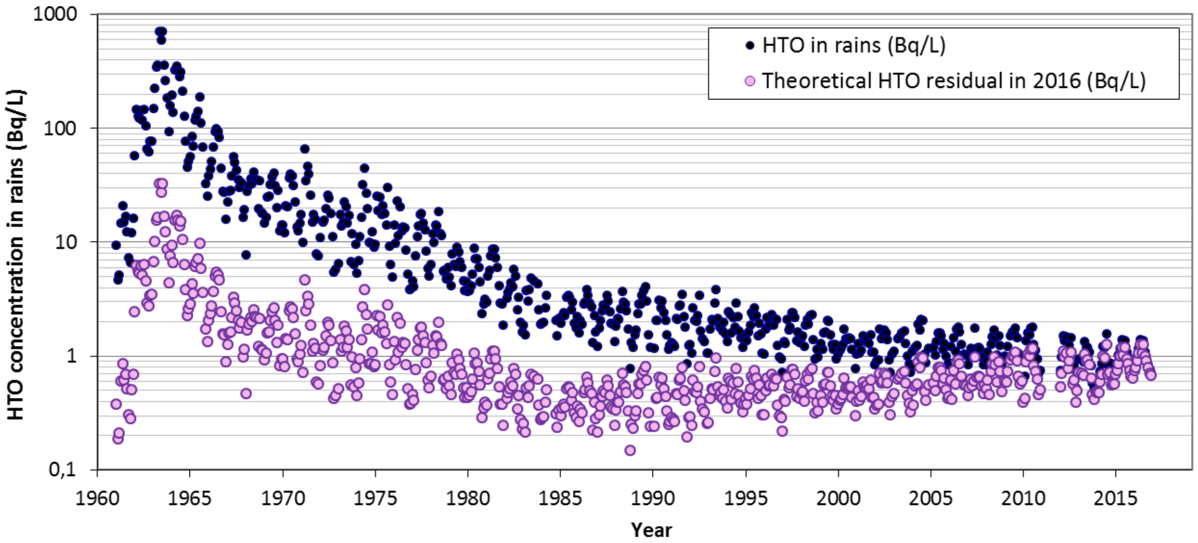

Figure B: Measured HTO levels in rain (Bq/L) and theoretical HTO residual contents in 2016 (Bq/L, pink points) from rain falls after radioactive decay account (Vienna, Austria, from IAEA Wiser data base, https://nucleus.iaea.org/Pages/GNIPR.aspx).

REFERENCES

1. Duffa, C. Répartition du plutonium et de l'américium dans l'environnement terrestre de la basse vallée du Rhône. Doctoral thesis, Aix Marseille University, France (2001).
